# Determinants of the evolutions of behaviours, school adjustment and quality of life in autistic children in an adapted school setting: an exploratory study with the International Classification of Functioning, disability and health (ICF)

**DOI:** 10.1186/s12888-022-03924-0

**Published:** 2022-05-06

**Authors:** Stéphanie Schneider, Céline Clément, Marc-André Goltzene, Nicolas Meyer, Agnès Gras-Vincendon, Carmen M. Schröder, Romain Coutelle

**Affiliations:** 1 Service de Psychiatrie de l’Enfant et de l’Adolescent, FHU NEUROGENΨCS, Fédération de Médecine Translationnelle de Strasbourg (FMTS), University Hospital and Medical School of Strasbourg, INSERM 1114, GIS Autisme et TND, Centre d’Excellence STRAS&ND, Strasbourg, France; 2 GIS Autisme et TND, Centre d’Excellence STRAS&ND, Strasbourg, France; 3 LISEC, Laboratoire Interuniversitaire en Sciences de l’Education et de la Communication, UR 2310, Université de Strasbourg, Strasbourg, France; 4 Hôpitaux Universitaires de Strasbourg, Service de pathologie professionnelle et environnementale, 1 place de l’hôpital, 67000 Strasbourg, France; 5 Department of Public Health, University Hospitals of Strasbourg, Strasbourg, France; 6 Faculty of Medicine, University of Strasbourg, Strasbourg, France; 7 CNRS UPR 3212, Equipe 9, “Lumière, Rythmes Circadiens, Homéostasie du Sommeil et Neuropsychiatrie”, Institut des Neurosciences Cellulaires et Intégratives (INCI), Strasbourg, France

**Keywords:** Autism Spectrum disorder, Quality of life, School adjustment, Evolution, International classification of functions disability and health (ICF), Day hospital

## Abstract

**Background:**

Previous studies about Quality of Life (QoL) in autistic children (ASD) have put forward the negative impact of factors such as Autism Spectrum Disorder (ASD) severity, psychiatric comorbidities and adaptive behaviour impairment. However, little is known about the relation of these factors to school adjustment, measured with the International Classification of Functions disability and health (ICF) framework (World Health Organization, 2001), and QoL evolutions. Thus, this study aimed at investigating the determinants of behaviours, school adjustment and QoL changes in 32 children in an ASD inclusion program over one academic year.

**Methods:**

Using Bayesian methods, we studied the impact of ASD severity, psychiatric comorbidities, adaptive behaviour level and a diagnosis of Pathological Demand Avoidance (PDA) on evolutions of behaviour, school adjustment (measured with the ICF) and QoL.

**Results:**

As predicted, adequate adaptive behaviour levels were associated with better progress of behaviours and school adjustment whereas psychiatric comorbidities were related to worse outcome of school adjustment. Contrary to our hypotheses, severe ASD was associated to better evolution of adjustment at school. PDA was not discriminant. We did not find any association between the studied factors and the evolution of QoL over the academic year.

**Conclusion:**

Our results show that the assessment of adaptive behaviour levels, psychiatric comorbidities and ASD severity level may be useful predictors to discriminate of school adjustment evolution (assessed by teachers within the ICF model) over a one-year period in autistic children. The assessment of this time course of school adjustment was sensitive to change and adapted to differentiate evolutions in an inclusive education framework. The investigation of quality of school life of autistic children as well as its determinants may therefore be relevant to improving academic adaptation. However, further research in larger groups, over longer periods and in different personalized school settings for autistic children is needed.

## Background

Autism Spectrum Disorder (ASD) is defined by communication-socialization impairments and restricted, repetitive patterns of behaviours [1]. The impact of this disorder is assessed, as per DSM-5, through three severity levels depending on the support required in daily life. However, the overall impact of ASD could be more deeply and globally studied through functioning and disability as described in the International Classification of Functioning, disability and health (ICF) [2]. The ICF model embraces body, individual and societal perspectives and aims at a best fit between the health condition, personal needs and social support. Finally, the ICF model can be used to assess quality of life (QoL) [3]. This has been previously done in youth with chronic conditions [4], in young people with haemophilia [5] and children with Duchenne muscular dystrophy [6].

Autistic children have lower QoL than their neurotypical peers [7–11]. The main risk factors for QoL impairment in autistic children are autism severity, behaviour problems, low adaptive behaviour level, and psychiatric comorbidities [8, 12]. Among these psychiatric comorbidities, ADHD [13] and anxiety disorder [14] are particularly detrimental. Qol impairment in ASD also involves parental well-being [7, 12, 15–17] which may be related to the child’s behaviour disturbances [18–21]. Finally, some Qol studies of autistic children investigated quality of school life. This investigation responds to the importance of school in children’s lives and follows the WHO recommendations [22]. Indeed, some studies have shown the relationship between students’ health and their school performance and satisfaction [23–26]. Quality of school life assessment is based on the use of classical scales such as the Kidscreen 27 [7] or the PedsQoL [12, 13, 27] with education sections, demonstrating impaired QoL at school. However, the assessment was very limited, with few items and not taking into account functioning, disabilities and associated factors compared to the ICF. This is why school adjustment of autistic children needs to be further investigated.

Adjustment that refers to “… the relative degree of harmony between and individuals needs and the requirements of the environment [28]” is a matter of concern in autistic children, especially at school. In ASD, school adjustment is mainly characterized by impairment in socialization and learning. Impairments in socialization are linked to isolation, lack of social reciprocity, social interaction impairments and poor friendship [29–32]. Moreover, autistic children show deficits in the social skills of cooperation, assertion and self-control as well as more hyperactivity and internalizing symptoms compared to the typically developing children [33]. Learning and academic achievement are overall impacted in ASD [34]. Learning disabilities can be associated with sensory disturbances [35] or cognitive impairments such as those involving calculation [36] and/or comprehension tasks [37].

The assessment of adjustment at school for autistic children has to take into consideration the high heterogeneity of the disorder [38] and of the educational environment. This heterogeneity is taken into account in DSM-5 [1] with specifiers such as intellectual impairment, language impairment, catatonia or presence of another neurodevelopmental, mental, or behavioural disorder, or the association with a known medical or genetic condition or an environmental factor. Another way to consider ASD heterogeneity is to investigate behavioural profiles. As such, the syndrome of “Pathological Demand Avoidance (PDA)” was coined to describe a profile of obsessive resistance to everyday demands and requests, with a tendency to resort to ‘socially manipulative’ behaviour, including outrageous or embarrassing acts [39, 40]. This term refers to a range of co-occurring behavioural difficulties frequently reported in autistic children but not limited to the autistic spectrum [41]. It remains controversial and is not included in the DSM-5 [1].

Previous studies about QoL in autistic children have linked QoL impairment, risk factors and adjustment difficulties at school. Key and redundant risk factors for QoL are ASD severity, comorbid ADHD, anxiety and adaptive behaviour levels [8, 12]. Previous studies in the field were mostly based on synchronic cross-sectional assessment even though the impact of these factors on evolution of outcome might be of special interest (diachronic longitudinal assessment). Thus, the aim of our study was to investigate, over one academic year, the impact of the above listed risk factors on evolutions of behaviour, school adjustment (measured with the ICF) and QoL in autistic children educated in an academic and therapeutic ASD program. In order to grasp ASD heterogeneity, we also investigated the impact of a PDA syndrome on these evolutions. Based on the current literature, we hypothesized that psychiatric comorbidities and severe ASD would be associated with poorer time course of behaviour, school adjustment and QoL in autistic children. In contrast, adequate adaptive behaviour levels were hypothesized to be associated with better evolutions of behaviour, adjustment at school and QoL. To the best of our knowledge, there is no study about QoL and the PDA syndrome in ASD. However, regarding the behavioural overlap between PDA and conduct disorders, the extremely high level of emotional problems [42] and their impact on family life [41], we postulated that PDA would be associated with poorer time course of behaviour, school adjustment and QoL in autistic children.

## Methods

### Participants

Thirty-two children and adolescents with ASD (ages 6 to 16 years) participated in the study (29 boys and 3 girls) (Table 1). This study took place during one academic year, from September 2016 to June 2017. The inclusion criteria were children admitted to the “therapeutic classes” program described below. After a full description of the study to parents, written informed consent was obtained for each child. Exclusion criteria were limited to parental opposition to the study. Among the 36 pupils admitted to the program, 4 were excluded because the parents refused or were unreachable.

**Table 1:** Clinical characteristics of children with ASD

	Children with ASD (*n* = 32)	
	Mean	(*SD*)
Age (years)	10.34	(2.70)
Sex (male) (N, %)	29	0.91
ADI-R^a^		
Social (cutoff: 10)	14.69	(5.62)
Communication (cutoff: 9)	11.65	(4.56)
Restrictive, repetitive behaviour (cutoff: 3)	4.56	(2.14)
SCQ^b^		
Total score	13.09	(6.32)
Severe autism (N, %)	4	13
Total IQ^c^	87.79	(17)
VABS-II^d^		
Daily living skills	69.71	(24.79)
Adequate adaptative level (N, %)	12	38
Moderately low and low adaptative level (N, %)	20	42
Mild intellectual disability (N, %)	8	25
Language disorder (N, %)	23	72
Genetic condition (N, %)	1	3
Neurologic comorbidity (N, %)	1	3
Perinatal history (N, %)	4	13
K-SADS^e^		
Anxiety disorder (N, %)	12	38
Generalized anxiety disorder (N, %)	5	16
Social phobia (N, %)	2	7
Specific phobia (N, %)	3	10
Separation anxiety disorder (N, %)	3	10
ADHD^f^ (N, %)	7	23
Anxiety disorder and ADHD (N, %)	4	13
EDA-Q^g^		
PDA (N, %)	6	19

^a^Autism Diagnosis Interview, Revised form

^b^Social Communication Questionnaire

^c^Intelligence Quotient

^d^Vineland Adaptive Behaviour Scale, 2nd edition

^e^Kiddie Schedule for Affective Disorders and Schizophrenia

^f^Attention-Deficit/Hyperactivity Disorder

^g^Extrem Demand Avoidance Questionnaire

The “therapeutic classes” [43, 44] program aims at providing intensive educational support to autistic children who have severe difficulties in a general school setting, even when assisted by support teachers to ease inclusion. Admission into this specialized program warrants a formal diagnosis of ASD [1]. Children with moderate or severe Intellectual Disability (ID) are not included in this school program. The “therapeutic classes” associate education in classical school and different therapeutic interventions in a child psychiatric unit. Both aspects, school and therapy, are closely interlinked. At school, the children are involved in an ASD academic program devoted to 6 to 16 years old pupils without or with mild ID, integrating for each subject either a general class or a specialized smaller size class, depending on their own needs and their profile of cognitive strengths and weaknesses. For example, if they succeed in maths they follow courses in their age-related general class. In contrast, if they deeply underscore in grammar they attended individualized courses in a specialised class. Within the child psychiatric unit, children receive therapeutic interventions aiming at enhancing reciprocal communication, socialization skills and preventing challenging behaviours. These interventions are provided by a multidisciplinary team involving speech and physiotherapists, psychologists, child psychiatric nurses, care-assistant, educators and child and adolescent psychiatrists. Thus overall, children are educated in an ordinary school but they receive individualized specific academic and therapeutic interventions related to their ASD cognitive and behavioural profile.

This study was approved by the local ethics committee (“Comité d’éthique des Facultés de médecine, d’odontologie, de pharmacie, des écoles d’Infirmières, de kinésithérapie, de maïeutique et des hôpitaux”), N° FC/dossier 2016–83.

### Procedure

All the children underwent two assessments, the first in September 2016 (time 1) and the second in June 2017 (time 2), at the beginning and at the end of the academic year. At time 1, the assessment included a diagnostic evaluation and the initial assessment of behaviour, adjustment at school and QoL. At time 2, the assessment consisted of a final evaluation of behaviour, adjustment at school and QoL. The assessments at time 1 and 2 were plurifocal and included parent, teacher and care team member reports. The scales completed at times 1 and 2 were all kept in a separate file which was not accessible throughout the study to the teachers, parents and care team members involved in the assessments.

#### Diagnosis assessment

The diagnosis of ASD was based on a standardized and pluridisciplinary assessment following the French recommendation for the diagnosis of ASD in childhood [45, 46]. The standard diagnosis tool used was the Autism Diagnostic Interview – Revised (ADI-R) [47].

As recommended in DSM-5 [1], we checked ASD severity and specifiers such as intellectual impairment, language delay, genetic condition, neurologic comorbidities (epilepsy) and psychiatric comorbidities.

The initial ASD severity was assessed with the current version of the Social Communication Questionnaire (SCQ) [48] validated in French [49]. The cut-off score is 22 for distinguishing severe from moderate and mild form of ASD. Informants were care team members.

Intellectual efficiency was assessed with the WISC-IV [50], WPPSI-III [51] or WNV [52] depending on the age and the profile of the children included. As recommended in DSM-5 [1], intellectual assessment must rely on intellectual functioning and adaptive functioning. Thus, adaptive behaviour was classically assessed with the Vineland Adaptive Behaviour Scale, Second edition (VABS-II) [53] validated in French [54]. Adaptive behaviour is related to communication, socialization and daily living skill. In our study, we took into account only daily living scales through the standard score and the corresponding adaptive level, i.e. adequate, moderate low and low.

We carefully explored the psychiatric comorbidities with the Kiddie-Schedule for Affective Disorders and Schizophrenia for school-age children (K-SADS) [55] validated in French [56]. Our investigations were limited to anxiety disorders and ADHD because of their high prevalence [57, 58] and their impact on QoL in ASD [13, 14]. The informants were the parents.

Finally, we used the “Extreme Demand Avoidance Questionnaire (EDA-Q)” designed to quantify PDA traits. This test has a good sensitivity (.80) and specificity (.85) [59]. In our study, informants were care team members. A score above 50 for children (5–11 years) and 45 for adolescents (12–17 years) is in favour of this syndrome.

#### Behavioural assessment

Behaviour in autistic children was assessed by parents and teachers with the corresponding report form of the Check Behaviour Checklist (CBCL) [60] validated in French [61]. The CBCL is a frequently used 111 items checklist designed to detect emotional and behavioural problem in children and adolescents. The questions are scored on a three-point Likert scale (0 = absent, 1 = occurs, 2 = occurs often). The applicability of the CBCL to autistic children has been supported by data on scale reliability, stability, and convergent validity [62, 63]. The total problems, total internalizing, and total externalizing scores were used.

Autistic features were assessed by care team members with the SCQ (see above, [48, 49]). ADHD features were assessed with the well-known parents’ and teachers’ versions of the Revised Conners’ Parent and Teacher Rating Scales (CRS) [64] translated in French [65].

Anxiety was assessed by parents with the parent version of the Screen for Child Anxiety Related Disorders (SCARED) [66] validated in French [67]. This scale is a 41 items, reliable and valid instrument to screen for childhood anxiety disorders in clinical settings [66].

#### Assessment of school adjustment

Adjustment at school was assessed with the “Support guide for autonomy assessment of pupils with special needs “(“*Guide d’aide à l’évaluation de l’autonomie des élèves reconnus handicapés*” in French) (GEVASco) [68].” This tool is based on ICF [69]. In the ICF, the term ‘functioning’ refers to all body functions, activities and participation, and ‘disability’ is similarly an umbrella term for impairments of function, limitations of activities and restrictions in participation. ‘Activity’ is defined here as the execution of a task or an action by an individual. ‘Participation’ is the involvement in a life situation. Activity limitations are difficulties an individual may have in executing activities whereas participation restrictions are problems an individual may experience when involved in specific life situations. Moreover, ICF lists environmental factors that make up the physical and social environment in which people live and conduct their lives.

The GEVASco assesses special needs for pupils with disabilities in France. It is a 32 items inventory. The items are related to five activities listed in the ICF. These activities are general tasks and demands (undertaking simple task….), interpersonal interaction and social relationships (formal and informal relationships…), mobility (using public buses…), self-care (putting on and/or taking off clothes…), communication (understanding simple sentences, non-verbal cues…). In GEVASco, the activities linked to general tasks and demands and social relationships merge. Moreover, this tool has a separate module that focuses on general tasks and demands specific to school (writing, reading, calculating…). The tool is completed by the teacher with the other involved professionals and the parents. In line with the ICF recommendations, teachers quote special needs on a four-level scale, activity performed without assistance and no difficulty, activity performed with mild assistance and/or mild difficulty, activity performed with assistance and difficulties, activity not performed. In order to study and quantify the responses we converted the levels in scores from 4 (no needs) to 1 (full needs). A score was created for each type of group of activities i.e. general task and demands and relationships, mobility, self-care, communication, general task and demands specific to school.

#### QoL assessment

QoL was assessed with the Kidscreen-27 [70, 71]. This scale investigates five dimensions: physical well-being (5 items), psychological well-being (7 items), autonomy & parents (7 items), peers & social support (4 items), as well as school environment (4 items). This scale has been used in previous studies on QoL in autistic children [7, 8].

### Data analysis

All the statistical analyses were performed under Bayesian paradigm and computed by using R version 4.0.5, OpenBUGS version 3.2.3 and all the required packages.

For this observational study, the sample size was determined pragmatically based on the number of children that attended the therapeutic classes being studied.

Descriptive analyses are expressed for numerical data as means ± standard deviations (SD) and categorical variables with frequencies and percentages.

Due to the repeated measures design of the study, the main outcome and most secondary outcomes were measured at the beginning and at the end of the follow-up period.

In order to study the global evolution between the beginning and the end of the study, we specified for each of the outcomes a linear regression model with a fixed time effect and a random effect per subject (to take into account the repeated nature of the data).

In order to study the specific evolution between the beginning and the end of the study according to the presence or not of a characteristic of interest, we specified for each outcome a linear regression model with a fixed time effect, a fixed characteristic effect, an interaction term between the time effect and the characteristic effect (in order to estimate a different temporal evolution according to the presence or not of this characteristic, which is our main interest) and a random effect per subject (to take into account the repeated nature of the data). The characteristic of interest were psychiatric comorbidities, Severe ASD, adequate adaptive level and PDA. The outcomes referred to behaviour, QoL and school adjustment outcomes. The behaviour outcome variables were the parent and teacher total CBCL score, the parent and teacher CRS score and the SCARED score. The QoL outcome variables were the five component scores of the Kidscreen-27. The outcome variables of school adjustment were the five dimension scores of the GEVASco.

Low informative priors (i.e., for each parameter, a normal distribution with mean = 0 and a variance = 1000) were used. The results are presented by the coefficients estimated by the model, their 95% credibility interval and accompanied by the a posteriori probability that this coefficient is strictly greater than 0. This probability thus refers: 1) for the time factor, to the probability of a difference between the initial time and the final time, 2) for the interaction term, to the probability of a differential evolution in the course of time according to the presence or not of the characteristic of interest. This probability must not be confounded with the usual *p*-value. A probability > 0.975 or < 0.025 was considered as statistically significant.

Computations were based on McMC samples of three chains with 50,000 iterations after a burn-in of 10,000 iterations and a thinning of 3. The chains convergence was checked graphically and with the RGB test and was observed in each case.

Bayesian methods were chosen because they are an appropriate alternative to the classical null hypothesis significance test (NHST) framework [72–75] Several papers also tackled scientists about the limitation of the NHST in health data [76, 77]. Finally, a recent publication underlines the numerous errors in using and interpreting the classical NHST [78] errors to which the Bayesian methods is partly a solution.

## Results

### Diagnostic evaluation at time 1

As expected, all the children had a valid diagnosis of ASD. Overall IQs levels, which were assessed with Wechsler scales (see above, [50–52]), ranged from 51 to 114 (SD = 17). Only one patient had an IQ total score below 55 (51) and could therefore be suspected of moderate intellectual disability but his profile was highly heterogeneous and his total IQ score was not representative. Moreover, his adaptive profile was respected (adequate level). We thus classified him in the mild intellectual disability range. 25% of children had a comorbid mild ID. 38% of children had an adequate adaptive level measured by the VABS-II (Table 1).

The frequency of anxiety disorders and ADHD, as assessed through the K-SADS, were 39 and 23%, respectively, with 13% of the children having both comorbidities. Finally, 19% of autistic children fulfilled the criteria of a PDA syndrome as assessed by the EDA-Q (Table 1).

Regarding pharmacological treatments, half of the included children received a psychotropic medication: methylphenidate (16%), risperidone (19%), aripiprazole (3%), cyamémazine (3%) and/or melatonin (6%).

### Evolutions of behaviours, adjustment at school and QoL from time 1 to time 2

The changes of behaviour, adjustment at school and QoL from time 1 to time 2 are presented in Table 2. Significant progress was noted in communication subscores of the SCQ evaluated by care team members, CRS scores reported by parents and teachers and SCARED scores, as well as regarding the total, externality and internality scores of CBCL (for teacher report only). Thus, communication impairment, ADHD and anxiety symptoms significantly decreased over one academic year.

**Table 2:** Changes in behaviour, adjustment at school and quality of life of children with ASD over one academic year

	Children with ASD at time 1 (*n* = 32)		Children with ASD at time 2 (*n* = 32)		Probability (time 2 > time 1)
	Mean	(*SD*)	Mean	(*SD*)	
SCQ^a^ (total score)	13.09	(6.32)	11.50	(6.50)	0.031
Reciprocal social interaction	4.25	(2.78)	3.69	(3.07)	0.104
Language/communication	4.66	(2.43)	3.56	(2.30)	< 0.01^f^
Restrictive, repetitive, stereotyped behaviour and interests	3.41	(2.21)	3.69	(2.06)	0.826
SCARED^b^ (parent report)	21.97	(11.75)	18.37	(9.33)	< 0.01^f^
CRS^c^ (parent report)	10.97	(6.09)	8.19	(4.39)	< 0.01^f^
CRS (teacher report)	13.84	(7.88)	8.31	(6.48)	< 0.01^f^
CBCL (parent report)					
Total score	54.26	(21.63)	52.03	(16.74)	0.230
Internalizing	15.90	(7.93)	15.26	(7.06)	0.11
Externalizing	11.81	(7.90)	10.89	(5.80)	0.44
CBCL^d^ (teacher report)					
Total score	77.31	(33.07)	32.19	(20.04)	< 0.01^f^
Internalizing	20.16	(8.45)	7.81	(5.35)	< 0.01^f^
Externalizing	18.47	(13.02)	7.00	(8.73)	< 0.01^f^
GEVASco^e^ (teacher report)					
General tasks and demands related to others	20.37	(3.96)	24.34	(5.31)	> 0.99^f^
Mobility	6.28	(1.69)	7.13	(2.87)	0.96
Self-care	11.03	(2.52)	11.97	(2.44)	0.438
Communication	11.00	(3.68)	12.19	(2.99)	> 0.99^f^
General tasks and demands related to school	25.06	(6.10)	32.50	(6.52)	> 0.99^f^
Kidscreen-27 (parent report)					
Physical well-being	16.48	(3.78)	15.96	(3.36)	0.29
Psychological Health Summary Score	26.42	(3.81)	26.22	(4.29)	0.46
Autonomy & parents	22.93	(5.11)	22.41	(5.39)	0.19
Peers & social support	8.84	(4.02)	9.26	(3.96)	0.68
School environment	13.42	(3.07)	13.89	(2.14)	0.69

^a^Social Communication Questionnaire

^b^Screen for Child Anxiety and Related Disorders

^c^Child Behaviour Checklist

^d^Revised Conners’ Parent and Teacher Rating Scales

^e^Support guide for autonomy assessment of pupils with special needs

^f^Probability higher than 95% or lower than 5% indicating a relevant difference between groups

**Fig. 1:**
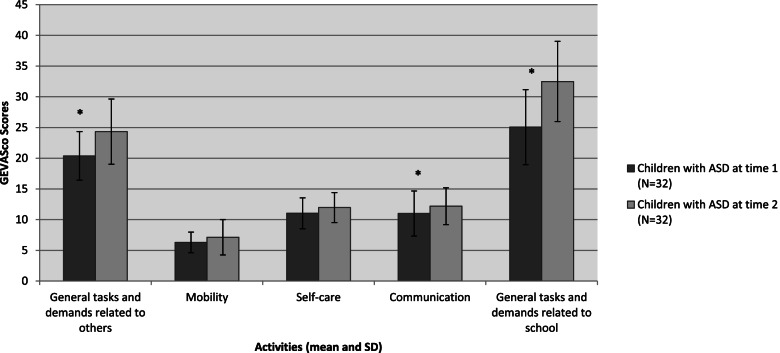
Mean and Standard Deviation (SD) activities scores measured with the GEVASco at time 1 and 2. Asterix indicates Probability higher than 95% or lower than 5% corresponding to a relevant difference between groups

The trajectory of adjustment at school, assessed by teachers, was characterized by higher performances in general tasks and demands, specific or not specific to school. Communicational abilities were also higher (Fig. 1.). Thus, communication skills and general tasks and demands improved over the academic year.

No significant changes in QoL, measured with the Kidscreen-27 components, were observed all over the year.

### The impact of ASD severity, psychiatric comorbidities, adaptive level and PDA diagnosis on the evolution of behaviours, adjustment at school and QoL

Severe ASD was associated with a better course of adjustment at school as measured by the GEVASco. The abilities involved were general tasks and demands related to school (β = 8.21, CI95% [2.19; 14.10], Pr = 0.99). We did not find any link between Severe ASD and behaviour or QoL outcomes.

In contrast, psychiatric comorbidities (anxiety disorder and/ or ADHD) were related to worse outcome in school adjustment as measured by the GEVASco. The domain involved was communication (β = − 2.58, CI95% [− 4.60; − 0.56], Pr = 0.007). We did not find any link between psychiatric comorbidities and behaviour or QoL outcomes.

Children with adequate adaptive level had better evolution of behaviour, as assessed with the CBCL by teachers (β = − 16.49, CI95% [− 31.08; − 1.53], Pr = 0.015) and with the CRS by teachers (β = − 7.78, CI95% [− 12.6; − 2.87], Pr = 0.0013). Finally, children with adequate adaptive level had a better time course of adjustment at school as measured by the GEVASco. The abilities involved were general tasks and demands and social relationships (β = 3.59, CI95% [0.48; 6.70], Pr = 0.98) and self-care (β = 2.48, CI95% [0.41; 4.56], Pr = 0.99). We did not find any link between adaptive level and QoL outcomes.

PDA diagnosis was not associated with any behaviour, QoL and school adjustment outcomes.

## Discussion

This study aimed to investigate the impact of severe ASD, psychiatric comorbidities, adaptive level and a PDA syndrome on the evolutions of behaviour, school adjustment and QoL in autistic children over one academic year. As predicted, psychiatric comorbidities in ASD children were associated with a worse time course of school adjustment. Furthermore, adequate adaptive levels were related to a better progress of behaviour and school adjustment. However, contrary to our predictions, severe ASD was associated with a better evolution of this adjustment. PDA was not associated with any specific evolution of behaviour, QoL or adjustment at school. We did not evidence any factor associated with QoL.

The negative impact of psychiatric comorbidities on the evolution of school adjustment echoes past studies showing [8, 12–14] that these comorbidities were associated to school QoL impairment. More ASD-like behaviours and higher anxiety severity predicts a lower school QoL [14]. Anxiety predicts also QoL impairment (including school QoL) in autistic children [79, 80] whatever the modality of report (self vs parental report) used. Moreover, in comparison of ASD alone, autistic children and ADHD have lower school functioning [13]. All these studies included larger ASD group (*N* = 65 to 3066) than our sample. However, none took into account the longitudinal impact of psychiatric comorbidities as we made. The link between adequate adaptive levels and a better progress of school adjustment concurs with other studies which highlighted the association between low adaptive level and QoL impairment [12, 81]. However, these studies did not specifically consider school QoL and the impact of adaptive level and QoL evolution. Our study therefore highlights the effect of psychiatric comorbidities and adaptive behaviour level on school adjustment in line with the literature in the field and completes our understanding of this effect through time.

The demonstration that psychiatric comorbidities and adaptive behaviour levels interact with the evolution of school adjustment in children receiving an ASD inclusion program, emphasizes how these factors may be assessed and taken into account in school programs geared toward children and adolescents with ASD. The importance of psychiatric comorbidities such as anxiety and ADHD in intervention has been previously shown [82, 83]. Indeed, the pioneers of the “Classes thérapeutiques” [43, 44] were aware of these comorbidities and linked the academic inclusion program with intervention in a child psychiatric outpatient unit. The impact of adaptive level has previously been highlighted and is considered a major target in ASD intervention [84, 85]. Unlike psychiatric comorbidities and adaptive behaviour, PDA was not discriminating. Our results are therefore in agreement with the authors who questioned the validity of PDA as a distinct entity [41].

Severity of ASD was associated with change in adjustment at school, but in the opposite direction of what was previously showed. Methodological differences may account for this result. We distinguished severe ASD by the SCQ (score above 22) when other studies used the Childhood Autism Rating Scale (CARS) [86], the Social Responsiveness Scale (SRS) [27], general psychopathological symptoms [12, 87] or adaptive behaviour levels [12, 87]. These discrepancies in assessment show how severe ASD can be seen either as a high level of autistic features or as a high level of disability. Thus, these two points of view can differ widely and lead to distinct results. Other studies included children with all ranges of ASD including those with moderate or severe form of ID [12, 86, 87] whereas our population consisted only of children without ID or with mild ID. Thus, both the population studied and the tools differed in our study compared to previous investigations. Another explanation of this unexpected result would be that, in our study, the most severe form of ASD corresponds to the more typical or “prototypical” form of ASD [88] whereas the less severe form would be the less typical. In consequence, this result might suggest that the ASD inclusion programs in which children were involved were more beneficial for the most typical form of autism.

We did not show any factors related to change in QoL as assessed by the KIDSCREEN-27 over the study period whereas we did find associations between the outcomes of school adjustment measured by GEVASco and four determinants (severe ASD, psychiatric comorbidities, adaptive level and PDA diagnosis). This discrepancy between QoL and adjustment at school assessment was unexpected because adjustment at school measured by the GEVASco within the ICF framework can be seen as a proxy of quality of school life since ICF was used to assess QoL in other disorders [4–6]. However, methodological differences may account for the discrepancy between the time course of QoL and adjustment at school. The KIDSCREEN-27 is a parent report whereas the GEVASco is completed by teachers. The KISCREEN-27 is a global appraisal of QoL through health, family, friends and school QoL, in contrast to the GEVASco which focuses only on quality of school life. In addition, the former assesses school QoL through only 4 items when the latter has 32 items.

Our study was exploratory because, first and to our best knowledge, no other study has ever explored the longitudinal impact of selected determinants on the evolution of behaviour, QoL and school adjustment in ASD. Second, our study was not designed to assess the efficacy of the “therapeutic classes” program. This study was highly ecological, using common tools and based on routine care. It was not an experimental setting. This type of study is especially relevant to the gap between practice and research in autism intervention [89]. Third, our study was exploratory in terms of the limited number of patients included, which was constrained by the context of the “therapeutic classes” in which the study took place. Fourth, we limited the period studied to one academic year. Therefore, our study was exploratory only and not confirmatory. Further research in larger groups, over longer periods with more than two assessments (in order to capture specific trajectories) and in different personalized school settings for autistic children is needed.

Our study has some significant strengths. First of all, to the best of our knowledge, this is the first study to test the relevance of the ICF model in the assessment of autistic children. Moreover, we highlighted the accuracy of teacher’s assessment with the ICF-Model to differentiate evolutions. Thus, the ICF-model emphasizes the variability of evolutions in autistic children and suggests, for example, specific interventions on psychiatric comorbidities or adaptive behaviours to ease inclusion. This result also may highlight the accuracy of teacher’s assessment as shown by Lerner et al. [90].

Our study is not without limitations. The number of children with ASD included is relatively small. Another limitation concerns observer bias in assessment. For example, teachers were involved in assessment and education of children and might be consequently prone to overestimate improvement. Thus, our results may not show the full efficiency of “therapeutic classes” even if an improvement in behaviour and adjustment at school has been measured between time 1 and time 2. Such an observer bias might also explain why some changes in behaviour (measured with the CBCL) were only observed by teachers and not parents, although both completed this scale. However, this discrepancy was far from systematic; both parents and teachers rated a decrease in ADHD symptoms using the same questionnaire (CRS) and, last but not least, teachers as well as parents and team care members were blind to our study hypotheses. This may have prevented observer bias in assumptions testing. Finally, our studied population was limited to autistic children without ID or with mild ID. Thus, our results cannot be extended to the full range of ASD.

## Conclusions

ASD severity, psychiatric comorbidities and adaptive behaviour levels were associated with the evolution of school adjustment as measured by teachers within the ICF framework. The assessment of this time course of school adjustment in autistic children was sensitive to change and adapted to differentiate evolutions in an inclusive education framework. Studying the quality of school life of autistic children as well as its determinants may therefore be relevant to improving academic adaptation. However, further research in larger groups, over longer periods and in different personalized school settings for autistic children is needed.

## Data Availability

The datasets generated and/or analysed during the current study are not publicly available due to French data protection laws but are available from the corresponding author on reasonable request.

## Abbreviations

ADI-R: Autism Diagnosis Interview, Revised form
ADHD: Attention-Deficit /Hyperactivity Disorder
ASD: Autism Spectrum Disorder
CBCL: Check Behaviour Checklist
CI: Credible Intervals
CRS: Revised Conners’ Parent and Teacher Rating Scales
EDA-Q: Extreme Demand Avoidance Questionnaire
GEVASco: Support guide for autonomy assessment of pupils with special needs
ICF: International Classification of Functioning, disability and health
ID: Intellectual Deficiency
IQ: Intelligence Quotient
K-SADS: Kiddie-Schedule for Affective Disorders and Schizophrenia for school-age children
PDA: Pathological Demand Avoidance syndrome
Pr: Probability
QoL: Quality of Life
SCARED: Screen for Child Anxiety Related Disorders
SCQ: Social Communication Questionnaire
VABS-II: Vineland Adaptive Behaviour Scale, Second edition
WISC-IV: Wechsler Intelligence Scale for Children—fourth edition
WNV: Wechsler Non-Verbal scale of ability
WPPSI-III: Wechsler Preschool and Primary Scale of Intelligence—third edition
